# Glycine-alanine dipeptide repeats spread rapidly in a repeat length- and age-dependent manner in the fly brain

**DOI:** 10.1186/s40478-019-0860-x

**Published:** 2019-12-16

**Authors:** Javier Morón-Oset, Tessa Supèr, Jacqueline Esser, Adrian M. Isaacs, Sebastian Grönke, Linda Partridge

**Affiliations:** 1 Max Planck Institute for Biology of Ageing, Joseph-Stelzmann-Strasse 9b, 50931 Cologne, Germany; 2 Department of Neurodegenerative Disease, UCL Institute of Neurology, Queen Square, London, WC1N 3BG UK; 3 Department of Genetics, Evolution and Environment, Institute of Healthy Ageing, University College London, Darwin Building, Gower Street, London, WC1E 6BT UK

**Keywords:** *C9orf72*, Dipeptide repeat proteins, PolyGA, *Drosophila*, Spread, Repeat size, Ageing

## Abstract

Hexanucleotide repeat expansions of variable size in *C9orf72* are the most prevalent genetic cause of amyotrophic lateral sclerosis and frontotemporal dementia. Sense and antisense transcripts of the expansions are translated by repeat-associated non-AUG translation into five dipeptide repeat proteins (DPRs). Of these, the polyGR, polyPR and, to a lesser extent, polyGA DPRs are neurotoxic, with polyGA the most abundantly detected DPR in patient tissue. Trans-cellular transmission of protein aggregates has recently emerged as a major driver of toxicity in various neurodegenerative diseases. In vitro evidence suggests that the C9 DPRs can spread. However, whether this phenomenon occurs under more complex in vivo conditions remains unexplored. Here, we used the adult fly brain to investigate whether the C9 DPRs can spread in vivo upon expression in a subset of neurons. We found that only polyGA can progressively spread throughout the brain, which accumulates in the shape of aggregate-like puncta inside recipient cells. Interestingly, GA transmission occurred as early as 3 days after expression induction. By comparing the spread of 36, 100 and 200 polyGA repeats, we found that polyGA spread is enhanced upon expression of longer GA DPRs. Transmission of polyGA is greater in older flies, indicating that age-associated factors exacerbate the spread. These data highlight a unique propensity of polyGA to spread throughout the brain, which could contribute to the greater abundance of polyGA in patient tissue. In addition, we present a model of early GA transmission that is suitable for genetic screens to identify mechanisms of spread and its consequences in vivo.

## Introduction

Frontotemporal Dementia (FTD) and Amyotrophic Lateral Sclerosis (ALS) are devastating and currently intractable neurodegenerative diseases, characterized histologically by the progressive loss of neurons in the frontal and temporal lobes, or upper and lower motor neurons, respectively [1]. Patients with either disease show a time-dependent progression of symptoms, yet the causes of this deterioration remain unknown.

An expansion of the hexanucleotide sequence GGGGCC in the *C9orf72* (C9) gene, ranging from 30 to several thousand repeats, is the most common familial cause for both FTD and ALS [2–4]. The hexanucleotide expansion is transcribed in both sense and antisense directions, and gives rise to hexanucleotide repeat RNA that accumulates in intranuclear and extranuclear RNA foci [2, 4–6]. In addition, the repeat RNAs can be translated in both directions in all reading frames, by repeat-associated non-AUG (RAN) translation, into 5 different dipeptide repeat (DPR) proteins: polyGA, polyGP, polyGR, polyPA and polyPR [7–9]. Numerous studies have addressed the differential toxicity of C9 RNA foci and DPRs, and have largely concluded that DPRs exert greater toxicity, especially the arginine-rich DPRs and, to a lesser extent, polyGA [10, 11]. However, the relative toxicity of the five DPRs has been mostly deduced from experimental production of proteins with much lower numbers of repeats than those seen in human patients, due to the difficulties in cloning repeat constructs. Importantly, the repeat length of proteins involved in other neurodegenerative diseases, such as huntingtin and ataxin-3, greatly influences their toxicity [12].

Although the arginine containing DPRs have so far proved the most toxic in animal and cellular models, in patients the contribution of different DPRs to overall toxicity is likely to be affected by their abundance. GA aggregates are the most abundantly detected DPR in patient tissues [8, 13], and it is therefore important to understand the behaviour of this protein at different lengths.

An emerging theme in the field of neurodegenerative diseases is that specific toxic proteins can spread trans-cellularly, thus contributing to the clinical progression shown by patients [14]. For instance, in experimental models, TDP-43, which typically aggregates in ALS and FTD [1], can spread trans-neuronally in cells [15] and mice [16]. Similarly, three independent studies have reported transmission of the C9 DPRs in cell culture models [17–19]. However, whether this phenomenon occurs in vivo remains unexplored.

We have investigated whether the C9 DPRs spread in vivo. We used the powerful genetics of *Drosophila* and found that, out of the three toxicity-associated DPRs, only GA DPRs spread in vivo in the fly brain, which accumulate in recipient cells as intracellular aggregate-like puncta. Furthermore, spreading was dependent on the repeat length of the GA DPRs, and their transmission was greater in the brains of older flies.

## Materials and methods

### *Drosophila* stocks and maintenance

Fly stocks were kept at 65% humidity on a 12:12 h light:dark cycle and fed a standard sugar/yeast/agar (SYA) diet [20]. For experiments using the pan-neuronal elav-GS driver, experimental flies developed and were allowed to mate for 2 days at 25 °C, after which female flies were sorted to SYA food with 200 μM RU486 (Mifepristone) at a density of 20 flies/vial and maintained at 25 °C for 3 days. Flies used for propagation experiments expressed the temperature-sensitive Gal4 inhibitor Gal80^ts^ to minimize the expression of the UAS transgenes during development. This inhibitor is active at 18 °C and can be inhibited to derepress Gal4 activity by shifting flies to 29 °C [21]. Therefore, flies used for propagation experiments developed and were allowed to mate for 2 days at 18 °C, after which female flies were sorted into SYA food at a fly density of 20 flies/vial and maintained at 18 °C or 29 °C as indicated for each experiment.

The following transgenic fly lines were obtained from the Bloomington *Drosophila* Stock Center: tubulin-Gal80^ts^ (BDSC_7019), orco-Gal4 (BDSC_23292), R9D03-Gal4 (BDSC_40726; hereafter referred to as OL-Gal4), UAS-eGFP.NLS (BDSC_4776) and UAS-syt.eGFP (BDSC_6926). The elav-GS driver line was obtained as a generous gift from Dr. Hervé Tricoire (CNRS, France) [22]. The rest of the fly lines used were generated for this study.

### Generation of transgenic fly lines and genetics

To generate the mCherry-tagged DPR constructs, we first PCR amplified mCherry using the Phusion polymerase (NEB) and the primers JOL13 and JOL14, which allowed for the addition of an N-terminal NotI restriction site (RS) followed by the linker GGTAGTGGAAGTGGTAGT, as well as a C-terminal KpnI RS after the stop codon. This amplicon was then ligated into the pUAST attB *Drosophila* transgenesis vector, thus forming the hereafter referred to as pUAST-mCherry-C plasmid. In parallel, we PCR amplified the sequences for GA36, GR36, PR36, GA100, GR100 and PR100 [10] using the TaKaRa LA Taq polymerase (Takara Bio Inc.) and the following primers: GA36fwd: JOL26; GA36rev: JOL33; GR36fwd: JOL26; GR36rev: JOL34; PR36fwd: JOL26; PR36rev: JOL35; GA100fwd: JOL26; GA100rev: JOL28; GR100fwd: JOL26; GR100rev: JOL30; PR100fwd: JOL26 and PR100rev: JOL30. This allowed for the addition of an N-terminal EcoRI RS followed by the ATG initiation site, as well as a C-terminal NotI RS. These amplicons were first ligated into the pBlueScript SK(+) plasmid for amplification and subsequently subcloned into the pUAST-mCherry-C plasmid. As a control, we also PCR amplified mCherry using the primers JOL9 and JOL14, which allowed for the addition of an N-terminal EcoRI RS followed by the ATG initiation site, as well as a C-terminal NotI RS after the stop codon. This amplicon was then directly ligated into the pUAST attB plasmid.

To clone the GA200 and GA200-mCherry constructs, we PCR amplified the GA100 sequence [10] in two independent reactions using the TaKaRa LA Taq polymerase and then ligated them together. First, we used primers JOL26 and JOL69 to add an N-terminal EcoRI RS followed by the ATG initiation site, as well as a SmaI RS and an XbaI RS at the C terminus. This amplicon was ligated into the pBlueScript SK(+) plasmid to obtain an EcoRI-ATG-GA100-SmaI-XbaI pBlueScript SK(+) plasmid. Second, we used JOL43 and JOL44 to add an N-terminal XbaI RS and a C-terminal stop codon followed by a NotI RS to GA100. Alternatively, we used JOL43 and JOL28 to add an N-terminal XbaI RS and a C-terminal NotI RS without a stop codon to GA100. The former was ligated into the EcoRI-ATG-GA100-SmaI-XbaI pBlueScript SK(+) plasmid to generate an EcoRI-ATG-GA100-SmaI-XbaI-GA100-Stop-NotI pBlueScript SK(+) plasmid, which was then subcloned into the pUAST attB plasmid (hereafter referred to as GA200). The latter was ligated into the EcoRI-ATG-GA100-SmaI-XbaI pBlueScript SK(+) plasmid to generate an EcoRI-ATG-GA100-SmaI-XbaI-GA100-NotI pBlueScript SK(+) plasmid, which was subcloned into the pUAST-mCherry-C plasmid, thus forming the hereafter referred to GA200-mCherry plasmid. To achieve high expression levels, all constructs contained the CACC Kozak sequence before their ATG initiation site. Finally, the sequence of all plasmids was verified by Sanger sequencing (Eurofins Genomics).

The sequences of all primers used for this study are included in Table 1.

**Table 1:** List of primers

Primer name	Primer sequence	Purpose
JOL13	ATATGCGGCCGCCGGTAGTGGAAGTGGTAGTGTGAGCAAGGGCGAGGAG	Generation of pUAS T-mCherry-C
JOL14	CCCCGGTACCTCACTTGTACAGCTCGTCCATG	Generation of pUAS T-mCherry-C and mCherry-only pUAST plasmids
JOL26	ATATGAATTCGGATCCCACCATG	Generation of GA36-mCherry, GR36-mCherry, PR36-mCherry, GA100-mCherry, GR100-mCherry and PR100-mCherry, GA200 and GA200-mCherry plasmids
JOL33	AAGCGGCCGCTGAAGCG	Generation of GA36-mCherry plasmid
JOL34	AAGCGGCCGCTGATCTGC	Generation of GR36-mCherry plasmid
JOL35	AAGCGGCCGCTGATCTGG	Generation of PR36-mCherry plasmid
JOL28	AAAAGCGGCCGCTGATGCTC	Generation of GA100-mCherry plasmid
JOL30	AAAAGCGGCCGCTGAACGTC	Generation of GR100-mCherry plasmid
JOL34	AAAAGCGGCCGCTGATCGAG	Generation of PR100-mCherry plasmid
JOL9	AAAAGAATTCCAACATGGTGAGCAAGGGCGAG	Generation of the mCherry-only pUAST plasmids
JOL69	CCGCGGCCGCTCTAGACCCGGGTGATGCTCCTGCTCC	Generation of the GA200 and GA200-mCherry plasmids
JOL43	GAATTCGGATCCCACCATGTCTAGAGGAGCT	Generation of the GA200 and GA200-mCherry plasmids
JOL44	CTTGCGGCCGCTTATGCTCC	Generation of the GA200 and GA200-mCherry plasmids
JOL28	AAAAGCGGCCGCTGATGCTC	Generation of the GA200 and GA200-mCherry plasmids

**Fig. 1:**
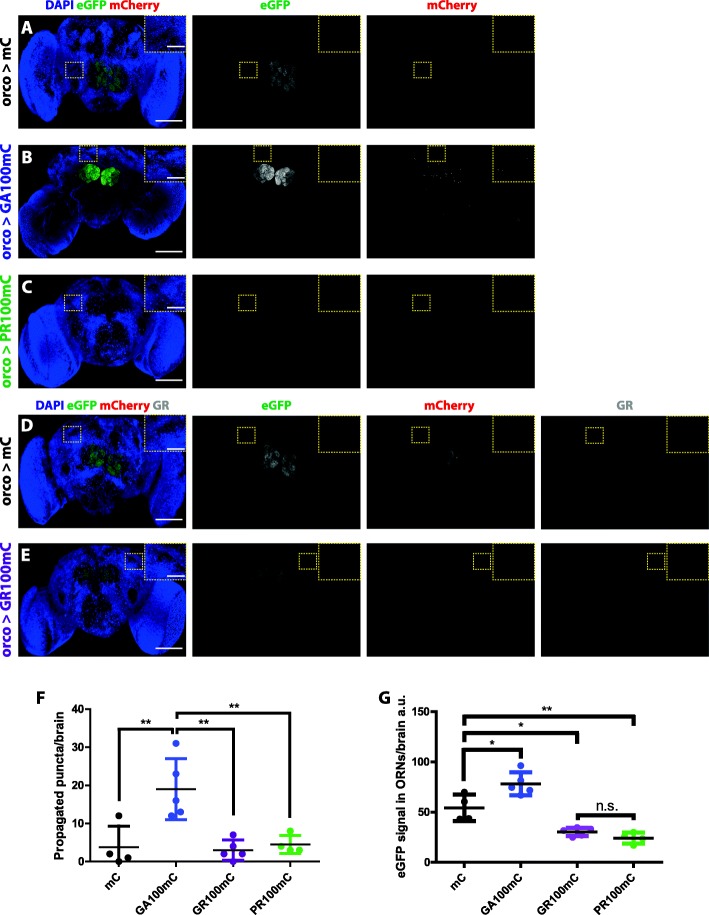
GA100-mCherry, but not GR100-mCherry or PR100-mCherry, can spread outside of ORNs. **a**-**e** Representative images of 5-days-old fly brains expressing mCherry (**a** & **d**), GA100-mCherry (**b**), PR100-mCherry (**c**) or GR100-mCherry (**e**) in Olfactory Receptor Neurons (ORNs) for 3 days. The same settings were applied to all genotypes while imaging their eGFP and mCherry signals. Spreading was only observed in flies expressing GA100-mCherry. EGFP and mCherry were detected using fluorescence as read-out. A GR-specific antibody was used to detect GR100-mCherry (**e**) and mCherry (**d**). Fly brains expressing only mCherry (**a** & **d**) were used as control to show that mCherry cannot spread by itself and to verify antibody specificity (**d**). Insets of the highlighted brain regions are shown. **f** Quantification of the number of mCherry puncta detected outside of ORNs across genotypes per brain after induction for 3 days. **g** Quantification of the eGFP signal detected within ORNs per brain (**P < 0.01 and *P < 0.05; One-way ANOVA, *n* = 4–6). Scale bars in images and insets are 100 um and 10 um, respectively

**Fig. 2:**
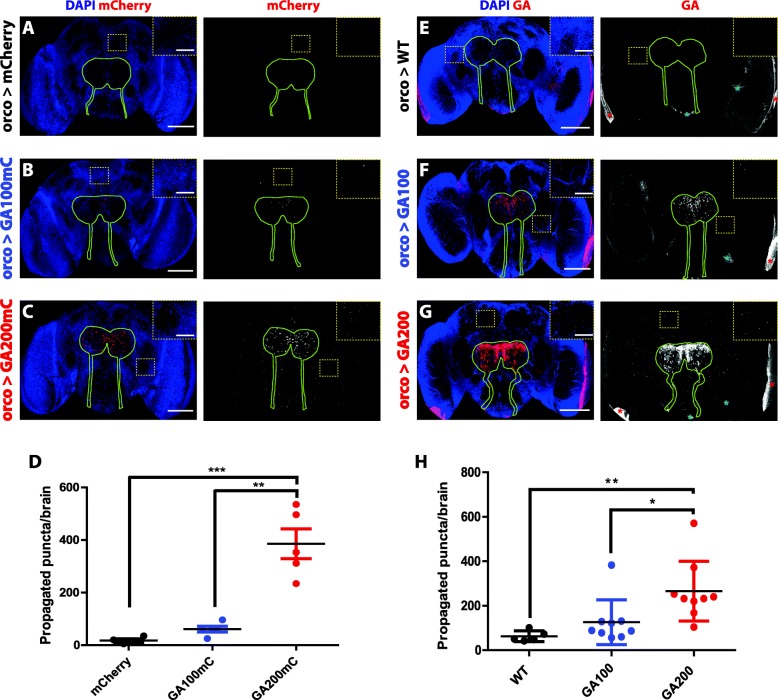
Spreading is increased in longer GA repeat proteins. **a**-**c** Representative images of 5-days-old fly brains expressing mCherry (**a**), GA100-mCherry (**b**) and GA200-mCherry (**c**) from Olfactory Receptor Neurons (ORNs) for 3 days. The same settings were applied to all genotypes while imaging their mCherry signals. Spreading is greater for GA200-mCherry than for GA100-mCherry. The mCherry signal was detected using its fluorescence as read-out. Flies expressing only mCherry (**a**) served as negative control to ensure that mCherry does not spread by itself. **d** Quantification of mCherry puncta detected in the central brain outside of ORNs across genotypes per brain after induction for 3 days (***P < 0.001 and **P < 0.01; One-way ANOVA, *n* = 4–5). **e**-**g** Representative images of 5-days-old fly brains expressing GA100 (**f**) or GA200 (**g**) in ORNs for 3 days and probed with an anti-GA antibody. GA200 spreads more than GA100. The same settings were used while imaging the GA signal across genotypes. Flies expressing only the driver (**e**) served to control for unspecific binding of the anti-GA antibody. Unspecific binding to trachea (blue asterisks) and the lamina of the optic lobes (red asterisks) was observed. **h** Quantification of GA puncta detected outside of the ORN boundaries across genotypes per brain after induction for 3 days. (**P < 0.01 and *P < 0.05; One-way ANOVA, *n* = 5–9). The boundaries of the ORN axons and synaptic terminals are highlighted with a solid green line. Insets of the indicated areas are shown to facilitate visualization. Scale bars in images and insets are 100 um and 10 um, respectively

**Fig. 3:**
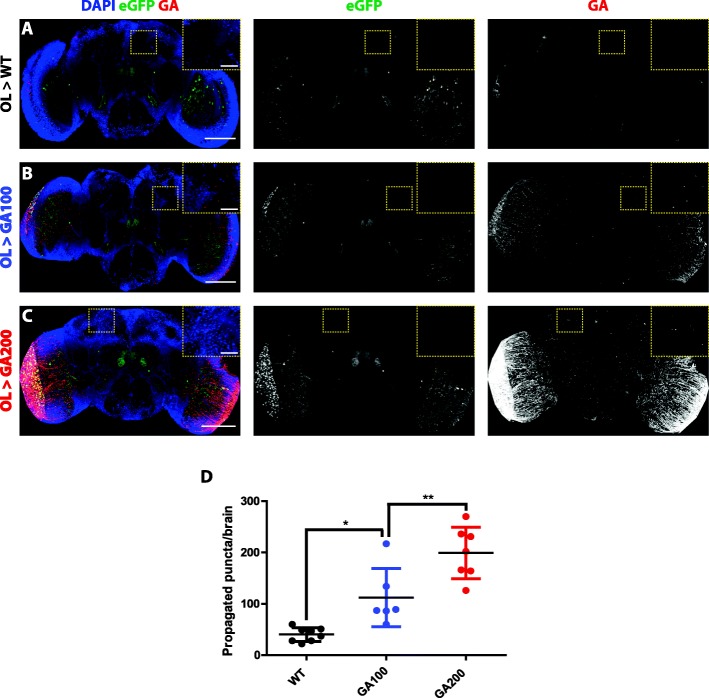
GA spreads in a repeat length-dependent manner from an independent neuronal population. **a**-**c** Representative images of 5-days-old fly brains from (**a**) control flies, expressing only the Optic Lobe (OL)-Gal4 driver, (**b**) flies expressing GA100 or (**c**) GA200 in the OLs for 3 days and probed with an anti-GA antibody. GA200 also spreads more than GA100 from this brain region. The same settings were used while imaging the GA signal across genotypes. EGFP with a nuclear localization signal was co-expressed to identify the cells targeted by the OL-Gal4 driver. **d** Quantification of GA puncta detected in the central brain outside of the targeted cells after expression of the indicated constructs for 3 days. Flies expressing only the driver (**a**) were used to control for unspecific binding of the anti-GA antibody (**P < 0.001 and *P < 0.05; One-way ANOVA, *n* = 6–8). Insets of the indicated areas are shown to facilitate visualization. Scale bars in images and insets are 100 um and 10 um, respectively

**Fig. 4:**
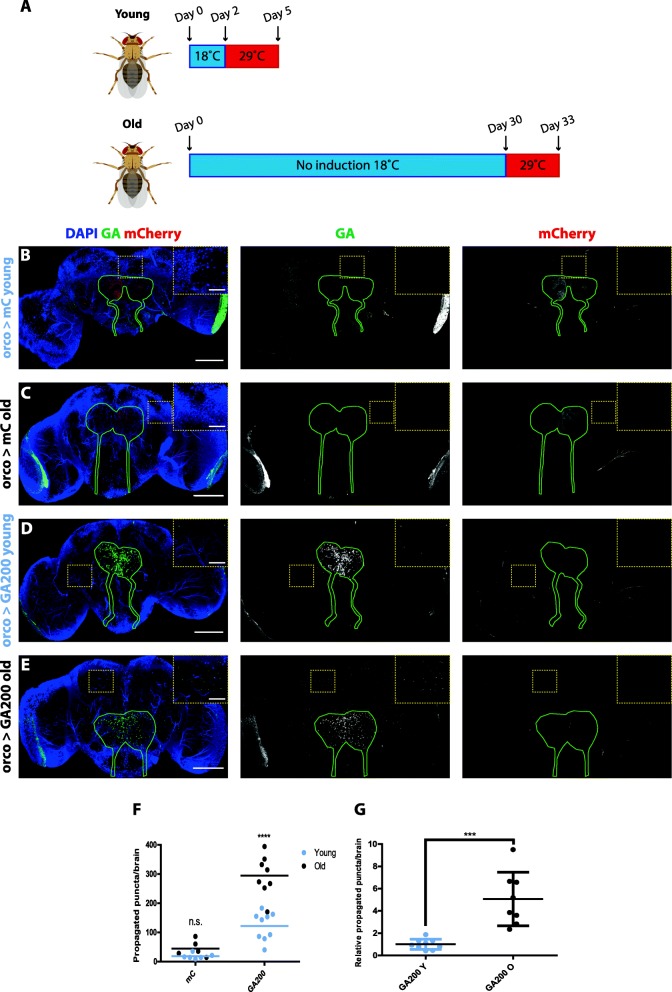
Age-associated factors exacerbate GA spread. **a**The expression of GA200 was induced for 3 days in Olfactory Receptor Neurons (ORNs) in young (2 days old) and old flies (30 days old), after which GA spread was measured. Fly cartoons were created with BioRender. **b** & **c** Representative images of control fly brains expressing mCherry in ORNs for 3 days in young (**a**) or old (**b**) flies. **d** & **e** Representative images of fly brains expressing GA200 in ORNs for 3 days in young (**d**) and old (**e**) animals. Brains were probed with an anti-GA antibody. The outline of ORN axons and synaptic terminals is shown in green. Insets of indicated areas highlight differences in the number of propagated dots across conditions. **f** Quantification of the total number of GA-positive dots detected outside of ORNs after 3 days of expression in young and old flies (age: ****P < 0.0001; genotype: ****P < 0.0001; interaction: ***P < 0.001; Two-way ANOVA, *n* = 5–9). **g** Quantification of the number of propagated GA-positive dots relative to the GA signal in ORNs after 3 days of expression in young and old flies (***P < 0.001, t-test). Scale bars in images and insets are 100 um and 10 um, respectively

Constructs were inserted into the fly genome using the phiC31 and attP/attB integration system [23]. For comparisons across the different DPRs, the landing site attP40 was used (i.e., Fig. 1 & Additional file 1: Figure S1, Additional file 2: Figure S2 and Additional file 3: Figures **S3**), whereas for comparisons across the different repeat lengths of GA the landing site attP2 was used (i.e., Figs. 2, 3, 4).

For all experiments, female Gal4 driver flies were crossed with UAS or wild-type (WT) male flies. To generate the final genotypes of the driver flies used for the propagation experiments, the orco-Gal4 and R9D03-Gal4 genes were recombined with UAS-syt.eGFP and UAS-eGFP.NLS, respectively. These flies were then crossed with tub-Gal80^ts^ flies and stable stocks were generated carrying the following genotypes: w^−^; w, tub-Gal80^ts^; w, orco-Gal4, w, UAS-syt.eGFP and w^−^; w, tub-Gal80^ts^; w, R9D03-Gal4, w, UAS-eGFP.NLS.

### Staining and imaging of adult *Drosophila* brains

Brains of adult female flies were dissected in PBS and immediately fixed in 4% paraformaldehyde at 4 °C for 2 h. Tissues were then washed 4–6 × 30 min in PBT (PBS with 0.5% Triton X-100) at room temperature (RT). For experiments where the mCherry and eGFP signals were imaged, brains were subsequently incubated in 50% glycerol in PBS for 1 h at RT after washing and mounted in VectaShield Antifade Mounting Medium with DAPI (Vectorlabs). For experiments where GA or GR were immunostained, brains were blocked in PBT with 5% fetal bovine serum and 0.01% sodium azide for 1 h at RT after initial washing and incubated with a mouse monoclonal anti-GA antibody (1:3000, Merck Millipore) or the 5H9 antibody against polyGR (1:50, [24]) overnight at 4 °C. Following 4–6 × 30 min washes in PBT at RT, brains were incubated with a suitable Alexa Fluor Secondary Antibody (Molecular Probes) overnight at 4 °C. Finally, brains were washed 4–6 × 30 min in PBT, incubated in glycerol-PBS and mounted.

To label cell membranes, brains were incubated in a rhodamine-conjugated phalloidin solution (Life Technologies) diluted in PBT at 0.2 U/ml for 15 min at RT. Brains were subsequently washed 3 × 30 min in PBT, incubated in glycerol-PBS and mounted.

Series of ﻿2-μm z-stacks across the whole fly brain were taken for each image using a Leica SP8-DLS confocal microscope and the same settings were used across genotypes and ages, unless otherwise stated. In experiments where DPR propagation was investigated, brains were imaged with settings where propagated puncta were over-exposed, both in the case of the GA100 and the GA200 constructs, and where the signal in the negative control, devoid of any DPR construct, was minimal. This was done in an attempt to maximize the detectability of signal. To further maximize the detectability of specific signal, HyD detectors, gating and the excitation wavelength that maximized the fluorescence emission of all fluorophores were used in all cases during imaging.

### Quantification of confocal images and statistics

All confocal images acquired for experiments where DPR spread was tested were first processed using ImageJ before subjecting them to quantification analysis. First, maximum z-stack projections were obtained to identify the lamina surrounding the optic lobes, as well as distinct artifacts, which were cropped from the stacks. In addition, areas of initial expression induction were also removed. For the latter, brain regions positive for eGFP were identified and cropped in experiments where eGFP was co-expressed along with the relevant DPR (i.e., Figs. 1, 3 & Additional file 3: Figure S3). This included the ORN axons and synaptic terminals for Fig. 1 and Additional file 3: Figure S3, or the medulla of the optic lobes, as well as a distinct region in the antennal lobes, when the OL driver was used. Alternatively, a rectangle spanning the visually detectable antennal lobes and the rest of the lower part of the central brain was drawn in experiments where eGFP was not co-expressed (i.e., Figs. 2 & 4), and its content was also cropped to ensure that no puncta within the axons or the terminals of ORNs were included in the quantification of propagated puncta. Puncta in the remaining brain areas were quantified from the cropped z-stacks in 3D using the image analysis software Imaris 9.2.0 ﻿ (Oxford Instruments). After background correction, the built-in spot detection algorithm was used to identify spots with a minimum size of 500 nm. Detection settings were adjusted based on the maximum intensity of the spots, which proved the most accurate filter to distinguish between strongly labelled spots (considered as real GA puncta) and weak/low quality spots from trachea or background. The same parameters were used for all of the conditions compared in the same experiment.

For the quantification of eGFP or GA levels in ORNS, we used their fluorescent signal in the ORN terminals in the antennal lobes as a proxy for their overall levels. Briefly, whole-brain stacks were taken with non-saturating settings for the ORN eGFP or GA signals, maximum intensity projections were generated from each z-stack, and the mean intensity of eGFP or GA in the synaptic terminals of ORNs was measured using ImageJ. ﻿The same settings were used for all of the conditions compared in the same experiment.

Statistical analysis was performed using GraphPad Prism. Individual statistical tests are indicated in the figure legends. Both One-way and Two-way ANOVA were always followed by Bonferroni post hoc test. *P* values < 0.05 were considered significant: *P < 0.05, **P < 0.01, ***P < 0.001 and ****P < 0.0001.

## Results

### GA DPRs, but not GR or PR DPRs, spread rapidly in the fly brain

To address whether toxicity-associated DPRs can spread in vivo, we generated novel fly lines that expressed mCherry-tagged GA, GR or PR with 36 or 100 repeats (hereafter GA36, GR36, PR36, GA100, GR100 and PR100) in a UAS-transgene. All transgenes were integrated into the same genomic locus, the attP40 landing site, which we previously confirmed to produce equal transcript levels of untagged DPRs [10]. mCherry-tagged DPRs were used in an effort to avoid differences in sensitivity of the different DPR-specific antibodies. To confirm expression of the DPR constructs, we generated flies with pan-neuronal induction of each of the mCherry-tagged DPRs, using the inducible elav-GS system, and imaged the mCherry signal in adult fly brains after induction for 3 days. Expression of all the DPR36-mCherry fusion proteins could be detected by imaging their mCherry signal (Additional file 1: Figure S1). The signal from the PR36-mCherry flies was stronger than that of GA36-mCherry and GR36-mCherry, probably due to the previously reported nuclear location of PR [25, 26]. However, while we detected the mCherry signal from mCherry-tagged GA100 and PR100 pan-neuronally (Additional file 2: Figure S2A-C), the presence of GR100-mCherry could only be verified when a GR-specific antibody was used (Additional file 2: Figure S2D-E). Interestingly, the expression of GR100-mCherry was almost exclusively detected in the median neurosecretory cells (MNCs) in the pars intercerebralis, where the expression levels of the majority of the DPRs tested, but not mCherry only, was also particularly high (Additional file 1: Figure S1 and Additional file 2: Figure S2). This suggests that MNCs may be particularly vulnerable to the accumulation of the C9 DPRs.

We next addressed whether the toxic DPRs have the ability to spread in vivo. Given that a previous study reported propagation of mutant huntingtin from Olfactory Receptor Neurons (ORNs) to other brain regions in *D. melanogaster* [27], we also initiated expression in this brain area. We imaged the brains of flies where ORN-specific expression of GA36-mCherry, GR36-mCherry or PR36-mCherry had been induced for 3 days in the adult fly using a temperature-inducible Gal80 and the ORN-specific orco-Gal4 driver [21, 28]. Since the cell bodies of ORNs are outside the central brain, and therefore only the axonal projections and synaptic terminals of ORNs can be detected in the adult central brain of *Drosophila* after dissection, we co-expressed eGFP-tagged synaptotagmin to label ORNs and control for driver specificity [29]. No specific mCherry signal was found outside of ORNs (Additional file 3: Figure S3), suggesting that the short isoforms of the toxic DPRs cannot spread, at least after short-term expression from this location. Interestingly, when flies were induced to express GA100-mCherry, GR100-mCherry or PR100-mCherry in ORNs for 3 days, mCherry-positive puncta were exclusively detected outside of the ORNs of GA100-mCherry-expressing flies (Fig. 1a-f), suggesting that longer GA DPRs are particularly prone to spread. Moreover, we detected a strong reduction in the eGFP fluorescence within the ORNs of flies expressing GR100-mCherry and PR100-mCherry (Fig. 1a-e, g), which may result from the well-known inhibitory effect of the arginine-rich DPRs on translation [30]. Also of note, unlike for GA100-mCherry, no specific mCherry signal was detected in the axons or synaptic terminals of ORNs in GR36-mCherry-, PR36-mCherry-, GR100-mCherry- or PR100-mCherry-expressing flies (Additional file 3: Figure S3C, D), indicating that these DPRs are not transported along axons, a potential requirement for DPR spread. Altogether, our data indicate that, out of the three toxic DPRs, at least for DPRs up to 100 repeats in length, only GA can spread from ORNs to the rest of the central brain.

### GA repeat length modulates the spread of GA DPRs

Unlike GA100-mCherry, GA36-mCherry did not spread, suggesting that spread is repeat length-dependent. To test this hypothesis further, we generated novel fly lines expressing mCherry-tagged GA100 and GA200 from the same genomic locus (attP2 landing site) to ensure equal transcript levels. In addition, to exclude the possibility that co-expression of eGFP-tagged synaptotagmin could influence transmission, we only expressed polyGA-mCherry. We measured the spread of the two mCherry-tagged constructs from ORNs, and found accumulation of mCherry puncta of both DPRs outside of this neuronal population after 3 days of expression induction, with substantially greater spread of the 200 than the 100 GA DPR (Fig. 2a-d). Spreading of GA was therefore more pronounced with longer repeats and was independent of eGFP-tagged synaptotagmin co-expression.

Since tags can interfere with protein function [31], we next tested the spread of untagged GA DPRs using GA100 and GA200 expressed in ORNs and a GA-specific antibody [32]. In agreement with our results using mCherry-tagged GA constructs, we found that the number of GA puncta detected outside of ORNs greatly increased with repeat length (Fig. 2e-h), further supporting the notion that the propensity of GA to spread is greater with longer GA repeats. To determine if GA could spread from different types of neurons, we tested whether untagged GA was transmitted from the optic lobes (OL). We expressed the GA constructs along with nuclear eGFP for 3 days in the OLs using the R9D03-Gal4 driver [33] and, consistent with our finding in ORNs, GA also spread in a repeat-length dependent manner from the OLs (Fig. 3a-d).

To determine if the propagated GA enters recipient cells, we co-stained brains from flies expressing GA200 in ORNs with fluorescently labelled phalloidin, a dye that strongly binds to actin F and can therefore be used to identify the boundaries of single cells in tissue [34]. Using this approach, we detected GA positive puncta in the cytoplasm of recipient cells, thus indicating that propagated GA puncta are intracellular (Additional file 4: Figure S4).

Altogether, our results show that, in two independent neuronal subsets, longer GA repeats spread in a length-dependent manner.

### GA DPRs exhibit an age-related increase in spreading

Given that ageing is a major risk factor for ALS and FTD [35], we investigated whether GA spread was affected by the age at which we induced GA expression. We induced ORN-specific expression of untagged GA200 starting in 2-day-old or 30-day-old adult flies for 3 days, and measured the spread outside of ORNs (Fig. 4a). There was a 3-fold increase in the total number of propagated GA puncta when GA expression was induced at the older age (Fig. 4b-f). Given that the accumulation of the peptides could change after expression induction at different ages, we also quantified the cumulative number of propagated GA puncta relative to GA expression in ORNs, as an indicator of whether the proportion of propagated GA compared to the total amount of GA in ORNs would change at different ages. Indeed, we found a larger proportion of propagated GA compared to ORN GA after expression induction in older brains (Fig. 4g), thus showing that the increased spread in older brains is not simply due to changes in the accumulation of GA in ORNs upon expression in older brains. Collectively, these results suggest that age-associated factors strongly affect the propagation propensity of GA DPRs.

## Discussion

In this study, we have explored whether the toxic C9 DPRs, namely GA, GR and PR, can spread, and the contributions of DPR repeat length and age to propensity to spread in vivo. Using the brain of adult *Drosophila* as a model, we show that GA, but not GR and PR, DPRs spread out of ORNs, at least as soon as 3 days after expression induction, and could therefore be a relevant early event in the pathogenesis initiation of C9 ALS and C9 FTD. In addition, while 36-repeat GA did not show evidence of spread, transmission occurred with expression of 100 and, to a greater extent, with 200 GA repeats. Finally, GA spread was more marked when its expression was induced at an older age.

Recent studies have shown that some proteins that typically aggregate in the brains of patients with various neurodegenerative diseases can be transmitted in model organisms. These findings have led to the hypothesis that protein transmission could underpin the clinical progression of such patients [14]. For instance, the clinical progression of ALS and FTD may be explained by the progressive spreading of TDP-43 pathology across conserved neuronal circuits relevant to these diseases [14, 16, 36, 37]. To investigate whether the toxicity-associated DPRs derived from a mutation in the *C9orf72* gene could also spread under in vivo conditions, we first used flies expressing, exclusively in ORNs, mCherry-tagged constructs of GA, GR and PR, to avoid an antibody bias, as antibodies specific for different DPRs could have different sensitivities. Interestingly, unlike mutant huntingtin, which strongly spread to a pair of large posterior neurons in the posterior protocerebrum upon expression in ORNs [27], the spreading pattern of GA DPRs was not specific to a single neuronal subset, either upon expression from ORNs or OLs, which might indicate that GA transmission does not only occur through synapses in vivo. By performing co-stainings with the cell membrane dye phalloidin, we found that the propagated GA signal accumulates in the shape of aggregate-like puncta inside recipient cells, which adds GA onto the growing list of proteins shown to spread from cell to cell in in vivo models of neurodegenerative diseases. In fact, GA accumulates in intracellular aggregates and is the most widely detected DPR in patient tissue [8, 13], which may be at least partially attributable to this ability to spread. Our result is also in line with a previous cell culture study, where GA, but not GR or PR, was found to spread from cell to cell [19]. Future studies should address what mechanisms are activated by GA, and not by GR or PR, that could be associated with its release from DPR-expressing neurons and/or its uptake by recipient neurons. While expression of the arginine-rich DPRs has been mostly associated with translation inhibition [38, 39], impairment of nucleocytoplasmatic transport [40–42], RNA processing [25, 43–45] and dysfunction of stress granule dynamics [39, 46], GA expression has been strongly correlated with impairment of proteostasis [47, 48]. Indeed, we observed that the co-expression of eGFP-tagged synaptotagmin with mCherry-tagged GR and PR led to decreased eGFP signal, which is likely due to their well-known inhibitory effect on translation [30]. In contrast, when we co-expressed mCherry-tagged GA, we found increased eGFP signal, suggesting that GA may be impairing eGFP degradation by damaging the proteostasis machinery. Therefore, we hypothesize that the preferential spread of GA could be associated with GA-induced proteostasis impairment, which has been shown to exacerbate the release of toxic proteins [49]. Another non-exclusive reason why GA, but not GR or PR, may spread could be related to the differential ability of neurons to transport each DPR along axons. In fact, unlike GA, we did not detect GR or PR in the axons or synaptic terminals of ORNs. Furthermore, GA, unlike GR and PR, has been shown to form oligomeric amyloids [17], which have been associated with greater propensity to spread [50]. This feature could also account for the greater propensity of GA to spread compared to GR and PR.

Unlike the distribution of phosphorylated TDP-43, which has been proposed to occur in patients in a progressive pattern indicative of pathology spread [36, 37], DPR aggregates have not yet been shown to occur in a staged manner. Thus, an argument against the relevance of DPR spreading is that they are produced by all cells expressing C9orf72, so do not necessarily need to receive an aggregate from a neighboring/connected cell to allow aggregate formation. However, it is possible that the addition of exogenous GA seeds could initiate aggregation of non-aggregated GA molecules, thus speeding up the process in receiver cells. How this might lead to TDP-43 changes is an open question but several possibilities exist. First, several studies have shown that GA itself can trigger cytoplasmic accumulation and hyperphoshorylation of TDP-43 [32, 51, 52]. Therefore, given that GA transmission would exacerbate the accumulation of GA, it could play a relevant role in eliciting the cytoplasmic accumulation and aggregation of TDP-43, which would then spread across the neuronal circuits relevant to ALS/FTD symptoms. This idea is supported by reports that the accumulation of GA aggregates precedes TDP-43 pathology [8, 53–55]. Second, one in vitro study showed that GA can spread within exosomes [18], which are known to comprise different kinds of biological material, including TDP-43 [56]. Therefore, GA accumulation could favour the formation of secretory vesicles, like exosomes, which would comprise, apart from GA aggregates, TDP-43 seeds or other material that would contribute to the degeneration of the ALS/FTD-relevant neuronal circuits. Indeed, the relevance of exosomes in the transmission of aggregation-prone proteins has already been shown in vivo [57]. Finally, while GA aggregates do not strongly correlate with neurodegeneration, or TDP-43 pathology [58], it cannot be excluded that specific forms of GA, e.g., oligomers or aggregates of a specific size, potentially not detected by inclusion staining, may correlate better with ALS/FTD neurodegeneration and TDP-43 pathology than the overall burden of GA aggregates. However, this point remains completely unexplored.

Both the morphology and spreading propensity of GA DPRs is dependent on repeat lengths. In the adult fly brain, GA36-mCherry shows a diffuse staining and does not spread. In contrast, GA100-mCherry and GA200-mCherry look aggregated and spread in a repeat length-dependent manner. These differences highlight the relevance of testing DPRs of different lengths to fully understand the behaviour of these peptides, and support future in-depth studies to understand the effect that repeat size has in the *C9orf72* mutation context in humans, which remains controversial [59–61] . In the case of the untagged GA DPRs, differences in GA transmission could be confounded by the GA antibody having more epitopes to bind to in the longer constructs, which would lead to stronger propagation signal being detected for GA200 than for GA100. However, this is unlikely because we also see greater spread for mCherry-tagged GA200 compared to mCherry-tagged GA100 when directly recording the mCherry fluorescence signal, which is only influenced by the overall abundance of each DPR as both GA100-mCherry and GA200-mCherry carry one mCherry tag per polyGA molecule. Given that the amino acid composition of GA100 and GA200 DPRs is the same, we hypothesize that the longer GA DPRs spread more either because they are more difficult to degrade, presumably due to their greater propensity to aggregate or their stronger effect on proteostasis, or because they earlier acquire a conformation that triggers their transmission. For instance, tau and α-synuclein, which typically aggregate and spread in Alzheimer’s disease and Parkinson’s disease models respectively, need to be at least partially aggregated to be able to propagate [14].

Ageing is a major risk factor for ALS and FTD [35]. We found that GA spread is greater when its expression is induced at a later age, suggesting that ageing-associated factors, such as impaired proteostasis, promote DPR spread, and therefore that GA transmission is likely to occur at a greater rate at old ages. These findings are in agreement with a recent study that reported a 2-fold increase in the spreading rate of tau upon injection of human tau-expressing viruses in aged murine brains compared to young ones [62]. For instance, GA aggregates may be degraded when they are formed at a young age, when the proteostasis machinery works efficiently, but accumulate during the progressive decline of proteostasis activity during ageing. The excessive accumulation of GA aggregates could trigger their mislocalization to exocytotic vesicles, thus mediating their extracellular release.

Taken together, we show that GA DPRs can rapidly spread in vivo, which is strongly influenced by their repeat length and the age of the GA-expressing neurons. The wide range of available tools to genetically manipulate flies makes this *Drosophila* model of early in vivo GA spread an attractive system that could be used in genetic or pharmacological screens to gain further insights into the mechanism(s) underlying GA transmission, its consequences for the recipient tissue and the search for interventions that can abolish this event.

## Conclusions

In conclusion, we have provided the first evidence for transmission of GA DPRs in an in vivo setting using the adult fly brain as a complex model. The extent of spread was magnified for longer GA DPRs and upon expression in old flies, suggesting that this mechanism could be of relevance for both the initiation and the progression of C9 ALS and C9 FTD.

## Supplementary information

**Additional file 1: Figure S1.** mCherry-tagged DPR36 constructs can be detected by imaging their endogenous mCherry signal. A-E show representative images of 5-days-old adult fly brains from flies induced to pan-neuronally express each of the indicated mCherry-tagged DPR36 construct for 3 days. 10 times lower settings were used to image mCherry (B) and PR36mCherry (E), as the signal was much stronger in those genotypes. For the rest of the genotypes, the settings were the same. No antibodies were used. Insets highlight the brain area where Median Neurosecretory Cells (MNCs) are located. Scale bars in images and insets are 100 um and 10 um, respectively.

**Additional file 2: Figure S2.** Detection of mCherry-tagged DPR100 proteins upon pan-neuronal expression. A-E Representative images of 5-days-old adult fly brains that pan-neuronally express the indicated mCherry-tagged DPR100 constructs for 3 days. 10 times lower settings were used to image GA100mCherry (B) and mCherry (D) as the signal was much stronger in those genotypes. No antibodies were used for A-C. D-E Fly brains were stained with an anti-GR antibody. GR100mC can be most clearly detected in the brain area where Median Neurosecretory Cells (MNCs) are located. Insets of the indicated areas are shown to facilitate visualization. Scale bars in images and insets are 100 um and 10 um, respectively.

**Additional file 3: Figure S3.** GA36-mCherry, GR36-mCherry and PR36-mCherry cannot spread from ORNs. A-D Representative images of 5-days-old fly brains expressing GA36-mCherry (B), GR36-mCherry (C) or PR36-mCherry (D) in Olfactory Receptor Neurons (ORNs) for 3 days. Synaptotagmin-eGFP was co-expressed in all genotypes to identify ORNs. Flies expressing mCherry (A) were used as a negative control to ensure that mCherry cannot spread by itself. No antibodies were used. Insets of the indicated areas are also shown to facilitate visualization. Scale bars in images and insets are 100 um and 10 um, respectively.

**Additional file 4: Figure S4.** GA propagated puncta are intracellular. A Representative image of a 5-days-old fly brain expressing GA200 in Olfactory Receptor Neurons (ORNs) for 3 days, and stained with an anti-GA antibody (green) and the rhodamine-conjugated fluorophore phalloidin (red). Scale bar = 25 um. B Inset of the area highlighted in a yellow dotted square in A outside of the ORN synaptic terminals where GA has propagated. Five cells positive for GA intracellular puncta can be observed. Scale bar = 3 um.

## Data Availability

All data generated or analysed during this study are included in this published article (and its supplementary information files).

## Abbreviations

ALS: Amyotrophic lateral sclerosis
C9orf72: Chromosome 9 open reading frame 72
DPR: Dipeptide repeat
eGFP: Enhanced green fluorescent protein
FTD: Frontotemporal dementia
GA: Glycine-alanine
GP: Glycine-proline
GR: Glycine-arginine
MNCs: Median neurosecretory cells
OL: Optic lobe
ORN: Olfactory receptor neurons
PA: Proline-alanine
PR: Proline-arginine
RAN: Repeat-associated non-AUG
RS: Restriction site
TDP-43: TAR DNA-binding protein of 43 kDa
WT: Wild-type
