# Supplementary figures and images for: Glycine-alanine dipeptide repeats spread rapidly in a repeat length- and age-dependent manner in the fly brain
Source: Acta Neuropathol Commun. 2019 Dec 16;7:209. doi: 10.1186/s40478-019-0860-x (PMC6916080; doi:10.1186/s40478-019-0860-x)

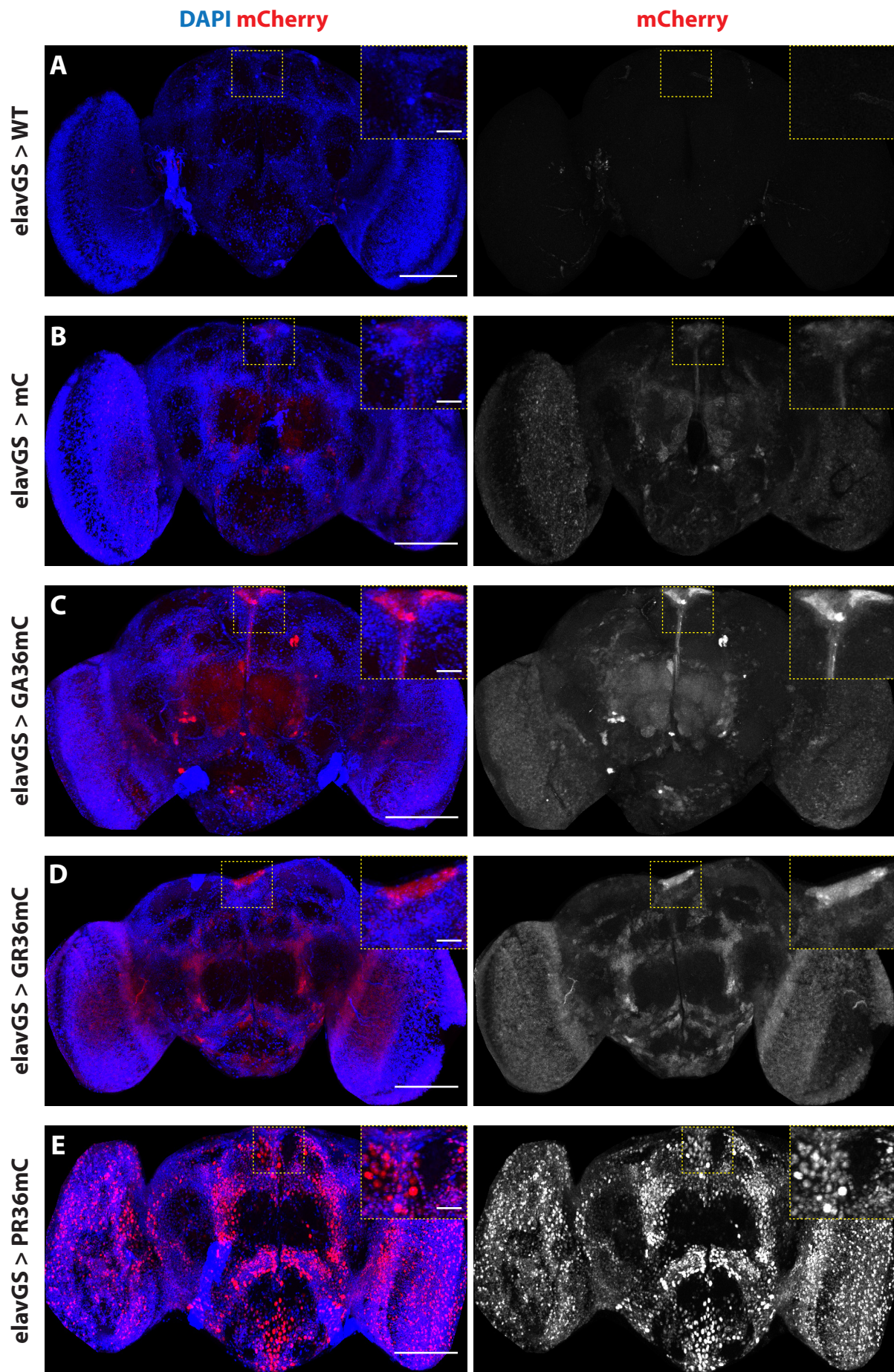

Supplement: Supplementary file 1 — Additional file 1: Figure S1. mCherry-tagged DPR36 constructs can be detected by imaging their endogenous mCherry signal. A-E show representative images of 5-days-old adult fly brains from flies induced to pan-neuronally express each of the indicated mCherry-tagged DPR36 construct for 3 days. 10 times lower settings were used to image mCherry (B) and PR36mCherry (E), as the signal was much stronger in those genotypes. For the rest of the genotypes, the settings were the same. No antibodies were used. Insets highlight the brain area where Median Neurosecretory Cells (MNCs) are located. Scale bars in images and insets are 100 um and 10 um, respectively. [file 40478_2019_860_MOESM1_ESM.pdf]

DAPI eGFP mCherry

eGFP

mCherry

orco > mC

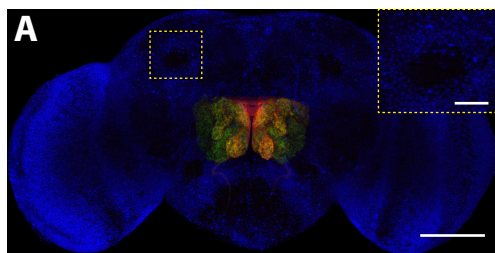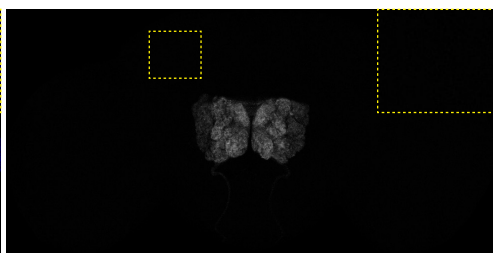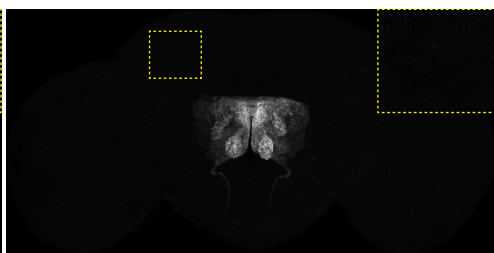

orco > GA36mC

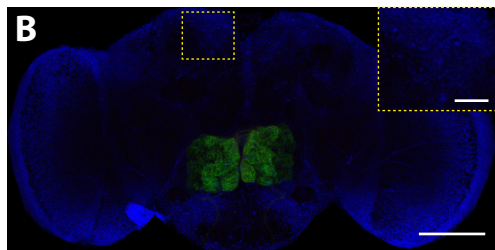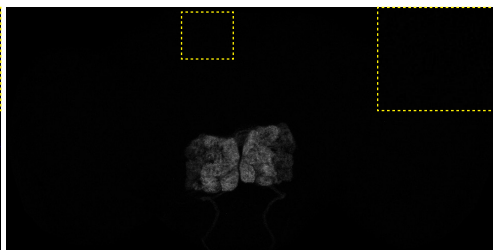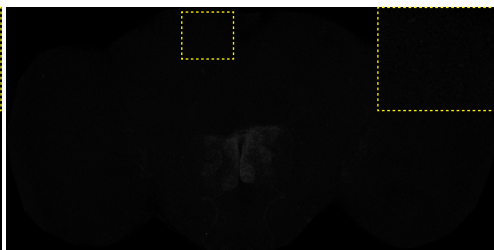

orco > GR36mC

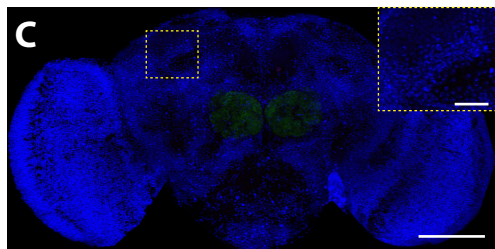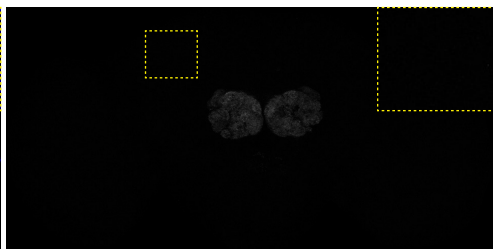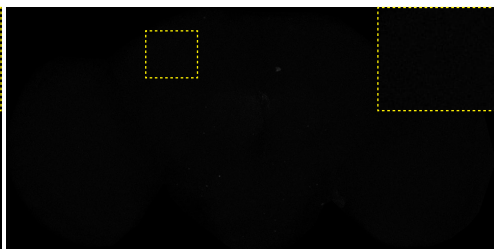

orco > PR36mC

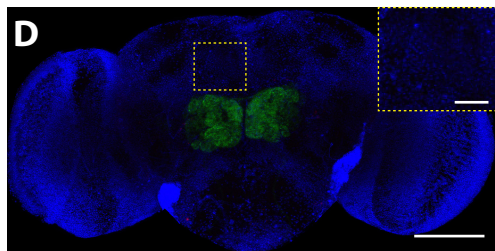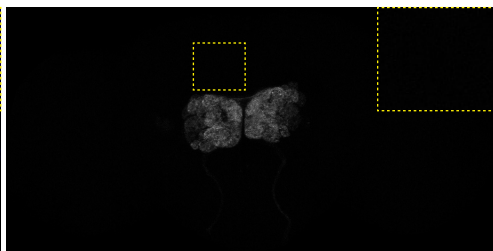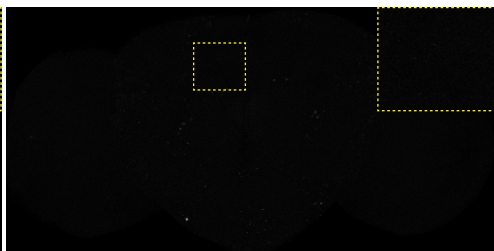

Supplement: Supplementary file 3 — Additional file 3: Figure S3. GA36-mCherry, GR36-mCherry and PR36-mCherry cannot spread from ORNs. A-D Representative images of 5-days-old fly brains expressing GA36-mCherry (B), GR36-mCherry (C) or PR36-mCherry (D) in Olfactory Receptor Neurons (ORNs) for 3 days. Synaptotagmin-eGFP was co-expressed in all genotypes to identify ORNs. Flies expressing mCherry (A) were used as a negative control to ensure that mCherry cannot spread by itself. No antibodies were used. Insets of the indicated areas are also shown to facilitate visualization. Scale bars in images and insets are 100 um and 10 um, respectively. [file 40478_2019_860_MOESM3_ESM.pdf]

DAPI GA Phalloidin

GA

Phalloidin

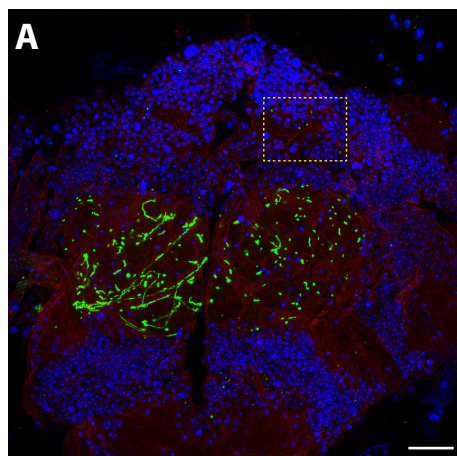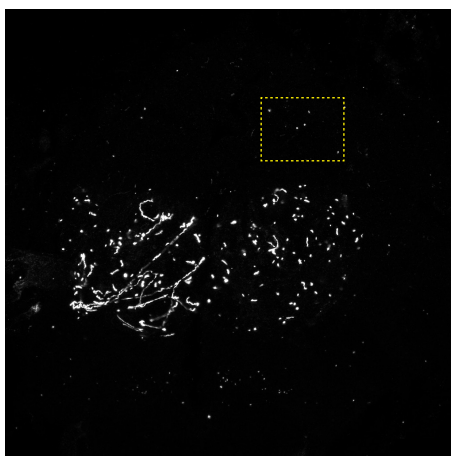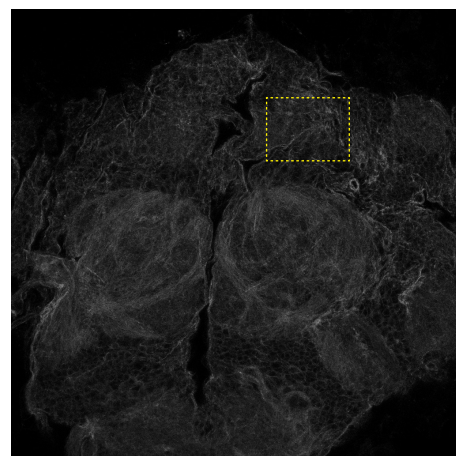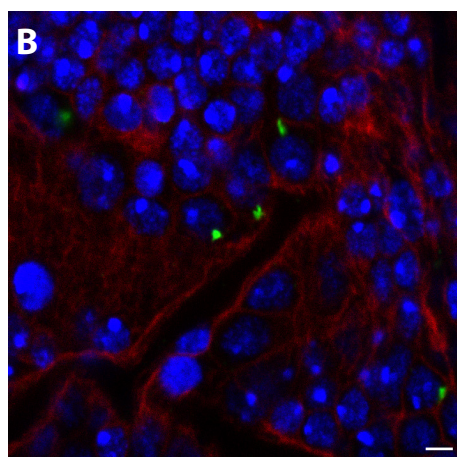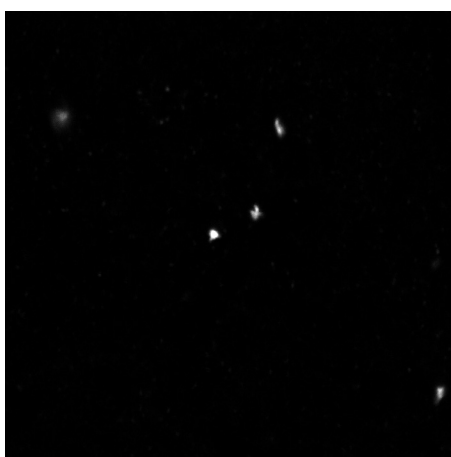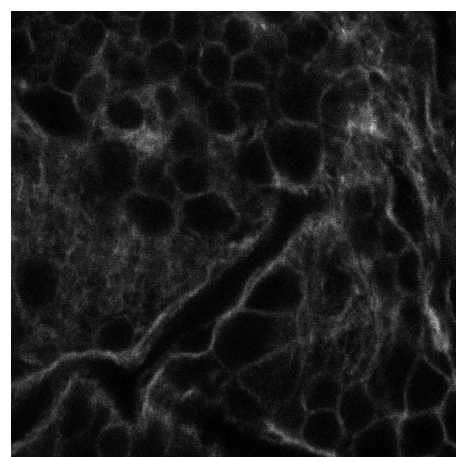

Supplement: Supplementary file 4 — Additional file 4: Figure S4. GA propagated puncta are intracellular. A Representative image of a 5-days-old fly brain expressing GA200 in Olfactory Receptor Neurons (ORNs) for 3 days, and stained with an anti-GA antibody (green) and the rhodamine-conjugated fluorophore phalloidin (red). Scale bar = 25 um. B Inset of the area highlighted in a yellow dotted square in A outside of the ORN synaptic terminals where GA has propagated. Five cells positive for GA intracellular puncta can be observed. Scale bar = 3 um. [file 40478_2019_860_MOESM4_ESM.pdf]
