# Supplementary material for: Glycine-alanine dipeptide repeats spread rapidly in a repeat length- and age-dependent manner in the fly brain
Source: Acta Neuropathol Commun. 2019 Dec 16;7:209. doi: 10.1186/s40478-019-0860-x (PMC6916080; doi:10.1186/s40478-019-0860-x)
Supplement: Supplementary file 2 — Additional file 2: Figure S2. Detection of mCherry-tagged DPR100 proteins upon pan-neuronal expression. A-E Representative images of 5-days-old adult fly brains that pan-neuronally express the indicated mCherry-tagged DPR100 constructs for 3 days. 10 times lower settings were used to image GA100mCherry (B) and mCherry (D) as the signal was much stronger in those genotypes. No antibodies were used for A-C. D-E Fly brains were stained with an anti-GR antibody. GR100mC can be most clearly detected in the brain area where Median Neurosecretory Cells (MNCs) are located. Insets of the indicated areas are shown to facilitate visualization. Scale bars in images and insets are 100 um and 10 um, respectively. [file 40478_2019_860_MOESM2_ESM.pdf]

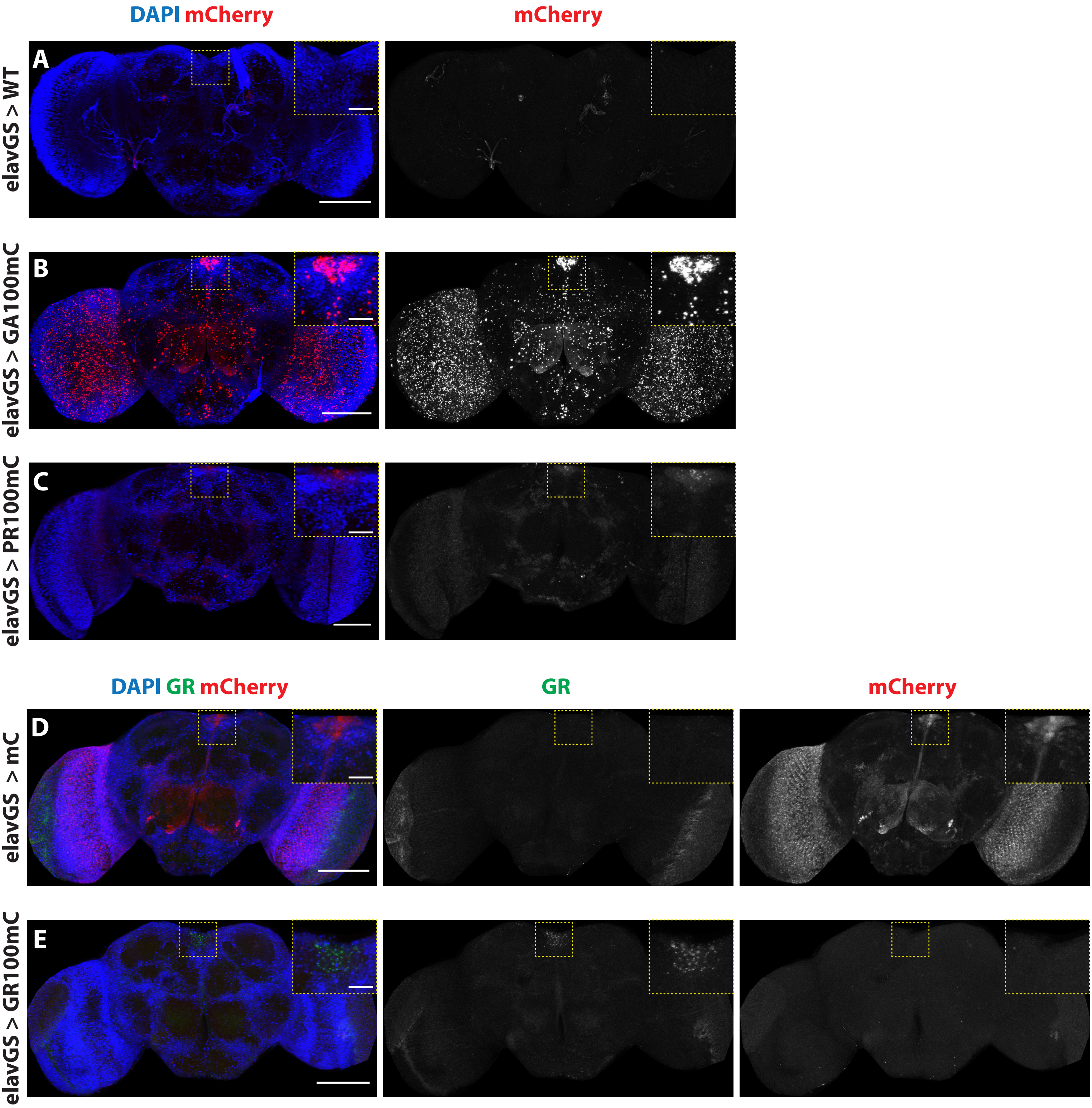
DAPI mCherry
mCherry
A
elavGS > WT
B
elavGS > GA100mC
C
elavGS > PR100mC
DAPI GR mCherry
GR
mCherry
D
elavGS > mC
E
elavGS > GR100mC
